# 10 Impact of the month of birth on the development of juvenile idiopathic arthritis among Tunisian children

**DOI:** 10.1093/rheumatology/keac496.006

**Published:** 2022-10-07

**Authors:** Wafa Triki, Emna Rabhi, Hanene Ferjani, Rania Ben Aissa, Dorra Ben Nessib, Kaouther Maatallah, Dhia Kaffel, Wafa Hamdi

**Affiliations:** 1 University Tunis el Manar, Tunisia, Faculty of Medicine of Tunis, Beb Saadoun, Tunisia; 2 Kassab Orthopedics Institute, Rheumatology Department, Ksar Said, Tunisia

## Abstract

**Background:**

Juvenile idiopathic arthritis (JIA) is the most common inflammatory disease influenced by genetic as well as environmental factors. Prior studies from Israel and USA suggested that JIA had a seasonality of birth, with birth peaking in winter and especially in January.

**Objectives:**

The aim of the study was to evaluate the impact of month of birth and season, and the onset of JIA in Tunisian children.

**Methods:**

We conducted a case-control study including 27 children with JIA compared with a homogeneous control group of 27 children hospitalized in a pediatric orthopedic unit for traumatic reasons and healthy for any chronic inflammatory rheumatism. Statistical differences between groups were also analyzed by non-parametrical tests.

**Results:**

Fifty-four patients (25 females and 29 males) were enrolled. The mean age was 11.04 ± 5.58 years and the mean duration of the diseases was 5.29 ± 3,18 years. The frequency of each JIA subset was at follows: polyarticular rheumatoid factor positive (*n* = 2), polyarticular rheumatoid factor negative (*n* = 5), psoriatic arthritis (*n* = 1), enthesitis-related arthritis (*n* = 11) and oligoarthritis (*n* = 8). Although the majority of children with JIA were born in November (22.2%) and December (18.5%), there was no significant difference in month of birth distribution between the cases and the controls. Likewise, there was no significant correlation between season of birth and the onset of the disease (*p* = 0,6).

**Conclusion:**

This study didn’t show any correlation between season and month birth, and the onset of JIA. Our result may be explained by the weather in Tunisia which is relatively warm with little variation in temperature between seasons compared with the country where they found a significant correlation. It also may be due to the limited number of our cases.

